# Tripartite motif containing 25 promotes proliferation and invasion of colorectal cancer cells through TGF-β signaling

**DOI:** 10.1042/BSR20170805

**Published:** 2017-07-12

**Authors:** Nianfeng Sun, Yu Xue, Ting Dai, Xiding Li, Nanxiang Zheng

**Affiliations:** Department of General Surgery, Nanjing Medical University Affiliated Wuxi Second Hospital, Wuxi City 214002, Jiangsu Province, China

**Keywords:** TRIM25, Colorectal Cancer, TGF-β signaling pathway, Proliferation, Migration

## Abstract

Tripartite motif containing 25 (TRIM25) is a member of TRIM proteins and functions as an E3 (ubiquitin ligase). It has been found to act as an oncogene in gastric cancer cells and is abnormally expressed in cancers in female reproductive system. Here, we investigated the function of TRIM25 in colorectal cancer. TRIM25 was found to be significantly up-regulated in colorectal cancer tissues and cancer cell lines through real-time PCR assay. Colorectal cancer cells (CRCs) overexpressing TRIM25 exhibited a two-fold higher proliferation and migration rate compared with their parental lines *in vitro*. Moreover, TRIM25 also promoted tumor progression *in vivo*. Further study indicated that TRIM25 worked through positively regulating transforming growth factor β (TGF-β) signaling pathway to regulate the proliferation and invasion of CRCs. In summary, our results indicate that TRIM25 also acts as an oncogene in colorectal cancer and it functions through TGF-β signaling pathway. Thus, TRIM25 represents potential targets for the treatment of colorectal cancer.

## Introduction

Colorectal cancer is the third most common cancer and the third leading cause of cancer deaths in men and women in the United States [1]. Colorectal cancer shows significant stage dependence of the 5-year overall survival rate [2]. The 5-year OS of patients without metastasis (the localized stage) can be more than 90%, while the 5-year OS of patients with distant metastasis at the time of diagnosis is less than 15% [1]. Colorectal cancer is undoubtedly a major health threat to world population. Despite recent advances in the screening and treatment of colorectal cancer, the prognosis of patients with colorectal cancer remains substandard [3]. The majority of colorectal cancer metastasis is to the liver, and surgical resection is the most effective therapy for liver metastases of colorectal cancer. However, metastatic recurrence following curative surgery is the leading cause of mortality [4,5]. Therefore, it is important to understand the biological mechanisms underlying colorectal cancer progression and identify the factors that contribute to the metastases of colorectal cancer.

Ubiquitination is a very important post-translational modification of proteins. Ubiquitination plays an important role in the regulation of cell proliferation, differentiation, and apoptosis [6]. It can signal for protein degradation via the proteasome, alter proteins’ cellular location, affect proteins activity, and promote or prevent proteins’ interactions [7–9]. Ubiquitination is carried out in three main steps: activation, conjugation, and ligation, performed by ubiquitin-activating enzymes (E1s), ubiquitin-conjugating enzymes (E2s), and ubiquitin ligases (E3s), respectively. Tripartite motif containing 25 (TRIM25, also known as estrogen-responsive finger protein) is a member of TRIM proteins and functions as an E3 ubiquitin ligase [10]. TRIM25 mediates K63-linked polyubiquitination of retinoic acid inducible gene I (RIG-I) and is critical for RIG-I-mediated antiviral signaling and interferon production [11]. Numerous viruses, including influenza A virus and Kaposi’s sarcoma-associated human herpes virus (KSHV), have evolved strategies to target this pivotal ubiquitination of RIG-I [12,13]. TRIM25 has also been broadly studied in the cancer area. It has been reported to be required for melanoma differentiation associated gene 5 (MDA5) and MAVS-mediated activation of nuclear factor κ-light-chain-enhancer of activated B cells (NF-κB) and interferon production [14]. In the past few years, TRIM25 has been found to be abnormally expressed in cancers of the female reproductive system. TRIM25 has an elevated expression in breast cancer [15,16] and ovarian cancer [17], but down-regulated in the endometrial carcinoma [18]. Overexpression of TRIM25 in lung cancer can regulate tumor cell progression [19]. It has also been reported that TRIM25 blockade by RNA interference inhibited migration and invasion of gastric cancer cells through transforming growth factor β (TGF-β) signaling [20]. However, the expression of TRIM25 in colorectal cancer and the connection of TRIM25 and colorectal cancer metastases have not yet been investigated. In the present study, we aim to investigate the function of TRIM25 in colorectal cancer *in vitro* and *in vivo*.

## Methods

### Colorectal cancer tissues

Colorectal cancer tissue samples were obtained from 11 patients who were diagnosed with colorectal cancer in Nanjing Medical University Affiliated Wuxi Second Hospital. Tumor samples were surgically removed and collected in separate tubes, then frozen by liquid nitrogen and stored at −80°C until use. The protocol was approved by the Committee on the Ethics of Animal Experiments of Nanjing Medical University Affiliated Wuxi Second Hospital (#WSH-jw87).

### Cell lines and mouse

Colorectal cancer cell (CRC) lines, HCT116 and HT29 were obtained from A.T.C.C. (Manassas, VA). Cells were maintained in RPMI-1640 medium (Invitrogen, Pleasanton, CA, U.S.A.), supplemented with 10% FBS (Invitrogen) at 37°C in 10% CO_2_. The nude mice were obtained from Nanjing Medical University. The present study was carried out in strict accordance with the recommendations in the Guide for the Care and Use of Laboratory Animals of the National Institutes of Health. The protocol was approved by the Committee on the Ethics of Animal Experiments of Nanjing Medical University Affiliated Wuxi Second Hospital (#WSH-ra03).

### Reagents

The following antibodies were used in the present study: anti-TRIM25, anti-actin, anti-TGF-β and anti-bone morphogenetic protein (BMP)-4 and purchased from Abcam (Cambridge, MA, U.S.A.). Anti-p-Smad2, anti-Smad2, anti-p-Smad4, anti-Smad4, and anti-tubulin were purchased from Cell Signaling Technology (Danvers, MA, U.S.A.).

### RNA extraction and real-time PCR

Total RNA was extracted from tissues and cell lines using TRIzol reagent (Invitrogen), according to the manufacturer’s instructions. Followed by real-time PCR as described recently [21], using GAPDH as an internal standard.

### Cell transfection

The HCT116 cells were plated into six-well plates with 2.5 × 10^4^ cells/well. Once the cells were 30–40% confluent, TRIM25 plasmid, accompanied with Lipofectamine 2000 (Invitrogen) were transfected into the HCT116 cells. The medium was changed after 24 h incubation.

### Cell proliferation assay

The HCT116 cells were seeded and transfected in six-well plates with 2.5 × 10^4^ cells/well, and subsequently transferred into 96-well plate with 3000 cells/well for 24 h following the transfection. The proliferation of the cells was detected using a cell counting kit-8 reagent (Dojindo Laboratories, Kyushu Island, Japan), according to the manufacturer’s instructions. The analysis was done as described recently [22], using a microplate reader (UV-6100; Shanghai Mapada Instruments Co., Ltd., Shanghai, China). The experiments were performed in triplicate.

### Lentiviral vectors and infection

Lentiviral vectors for TRIM25 overexpression were constructed by Shanghai Liangtai Biotechnology Company. The recombinant lentivirus and the negative control lentivirus were prepared and titered to 10^9^ TU/ml (transfection units). To obtain the stable cell lines, cells were seeded in six-well plates with 2 × 10^5^ cells/well. The cells were infected with the same viral titer with 8 μg/ml polybrene the following day. After 72 h of viral infection, the culture medium was replaced with selection medium containing 4 μg/ml puromycin. The cells were cultured for at least 14 days. The puromycin-resistant cell clones were isolated, amplified in medium containing 2 μg/ml puromycin for 7–9 days, and transferred to a medium without puromycin.

### Western blot

Cells were harvested and lysed in ice-cold RIPA lysis buffer (1% NP40, 0.5% Na-deoxycolic acid, and 0.1% SDS in PBS) with added fresh protease inhibitor (Sigma–Aldrich, St. Louis, MO, U.S.A.). The protein concentration was quantitated using the BCA protein assay from Pierce Bioscience (Vazyme E112-01/02, Nanjing, China). Whole-cell lysates with equal amount of protein were separated by SDS/PAGE and transferred on to PVDF membranes (Millipore, Bredford, MA, U.S.A.). Membranes were blocked with 5% skim milk and then incubated with primary antibodies at 4°C overnight. Membranes were visualized using the appropriate secondary antibodies at room temperature for 1 h followed by the ECL system (Pierce, Rockford, IL, U.S.A.) according to the manufacturer’s instructions.

### Mouse subcutaneous model

HCT116 cells were harvested at 70–80% confluence and counted. The nude mice were subcutaneously injected with 1 million HCT116 cells/mouse. Tumor volume was measured every week.

### Wound healing assay

Cells were cultured to 90–100% confluence in six-well plates and subsequently scratched using sterile pipette tips. After scratching, the wells were gently washed with medium to remove the detached cells, and then the medium without FBS was added. Scratched cells were photographed under an inverted microscope after 0, 8, 12, and 24 h. Migration of cells was evaluated by measuring the width of the scratched area at each time point. Each experiment was repeated at least three times.

### Cell transwell invasion assay

HCT116 cells on 12-well plates were transfected with TRIM25 expression plasmid or control plasmid. Forty-eight hours after transfection, cells were added to the upper chamber of transwell insert (8 mm pore size, Corning Costar, New York, NY, U.S.A.) with Matrigel-coated membrane (BD Bioscience, Franklin Lakes, NJ, U.S.A.). DMSO or 10 μM TGF-β inhibitor, SB431542 (Sigma–Aldrich, U.S.A.), was added to the upper chamber. Medium containing 20% FBS was added to the lower chamber. After 24 h, cells that did not invade through the pores were completely removed by a cotton swab. Invaded cells were fixed with 4% paraformaldehyde (Sigma–Aldrich, U.S.A.), stained with 0.2% Crystal Violet (Sigma–Aldrich, U.S.A.), and counted under a microscope. Each experiment was repeated at least three times.

### Statistical analysis

Data from three independent experiments were presented as the mean ± S.D. Two-tailed Student’s *t* test was used to calculate *P*-value, and *P*<0.05 was considered as statistically significant. Statistical analysis was carried out using GraphPad Prism (San Diego, CA, U.S.A.).

## Results

### TRIM25 is up-regulated in colorectal cancer tissues and cell lines

**Figure 1:**
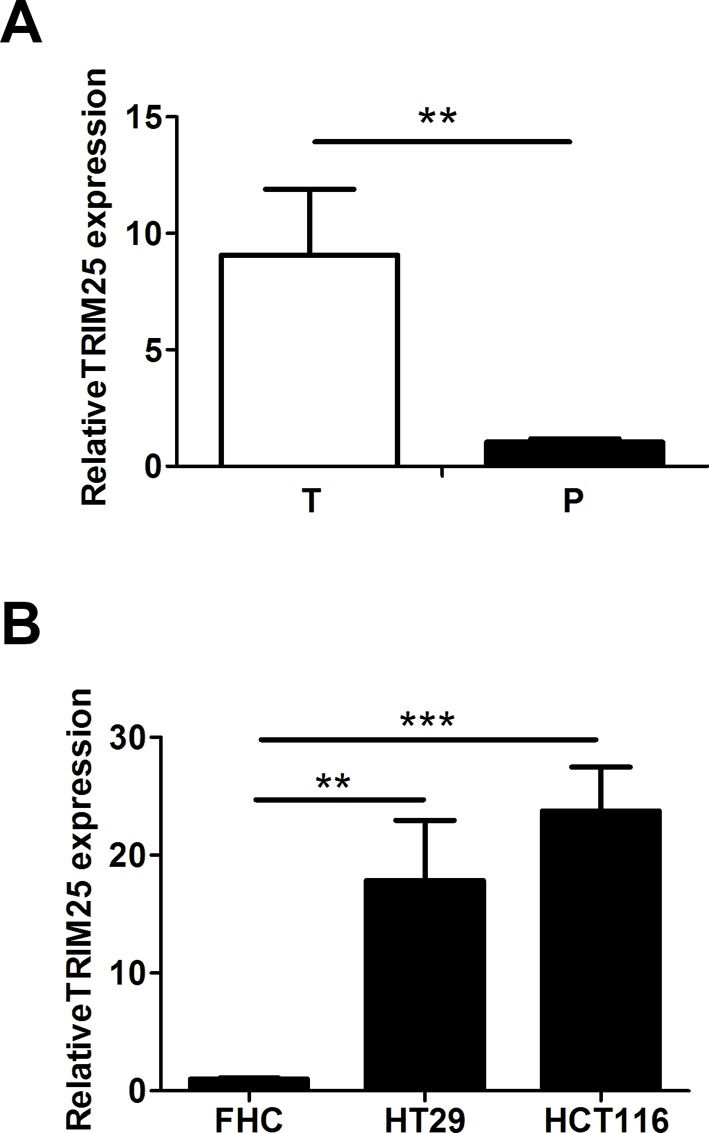
Higher expression of TRIM25 was observed in colorectal cancer tissues and cell lines. TRIM25 is up-regulated in colorectal cancer tissues and cell lines. (**A**) The relative expression of TRIM25 in human colorectal cancer tissues (*n*=11) and the corresponding adjacent tissues (*n*=11) was evaluated by real-time-PCR. P, corresponding adjacent tissues; T, colorectal cancer tissues. (**B**) The relative expression of TRIM25 in CRCs (HT29 and HCT116) and colonic epithelial cell line (FHC) was evaluated by real-time-PCR. **P<0.001, ***P<0.0001.

To determine the function of TRIM25 in colorectal cancer, we first examined the expression levels of TRIM25 in human colorectal cancer samples and their corresponding adjacent tissues by real-time PCR. The results showed that TRIM25 was significantly overexpressed in colorectal cancer tissues, compared with the corresponding adjacent tissues (Figure 1A).

To further verify TRIM25 expression in colorectal cancer, we then compared the TRIM25 expression levels between CRC lines and colonic epithelial cell line by real-time PCR. CRC lines (HT29 and HCT116) also have a significantly elevated expression of TRIM25 compared with the colonic epithelial cell line FHC (Figure 1B).

### TRIM25 promotes the proliferation of CRCs *in vitro* and *in vivo*

**Figure 2:**
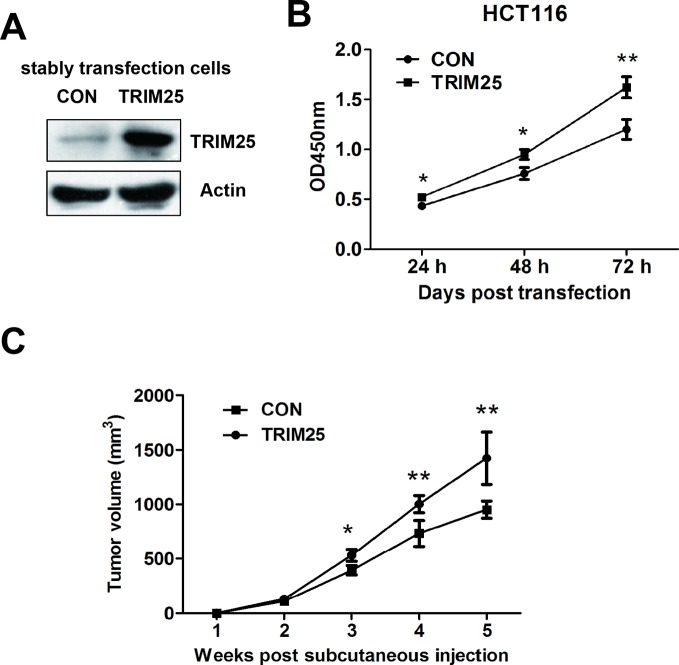
TRIM25 promotes the proliferation of CRCs both *in vitro* and *in vivo*. (**A**) Western blot assay of TRIM25 and action levels of HCT116 cells stably transfected with either control plasmid (CON) or TRIM25 overexpressing plasmid (TRIM25). (**B**) *In vitro* CCK-8 proliferation assay was performed to determine the proliferation rate of HCT116 cells transfected with either CON or TRIM25. (**C**) *In vivo* tumor progression assay of nude mice with subcutaneously injection of HCT116 cells stably transfected with either CON or TRIM25. Tumor volume was measured every week. *P<0.05, **P<0.001.

To assess the potential role of TRIM25 in colorectal cancers, TRIM25 or control plasmids were stably transfected into HCT116 cells (Figure 2A). A CCK-8 assay was subsequently used to evaluate the proliferation effects of TRIM25 in CRCs. As shown in Figure 2B, overexpression of TRIM25 significantly promotes HCT116 cells proliferation.

To further confirm that TRIM25 can stimulate cancer cell proliferation in colorectal cancers, *in vivo* tumor progression assays were performed using nude mice xenograft model. Following subcutaneous injection for 5 weeks, the tumor volume of the TRIM25 overexpression group revealed a significantly higher progression trend compared with the control plasmid group (Figure 2C).

### TRIM25 promotes the migration of CRCs

**Figure 3:**
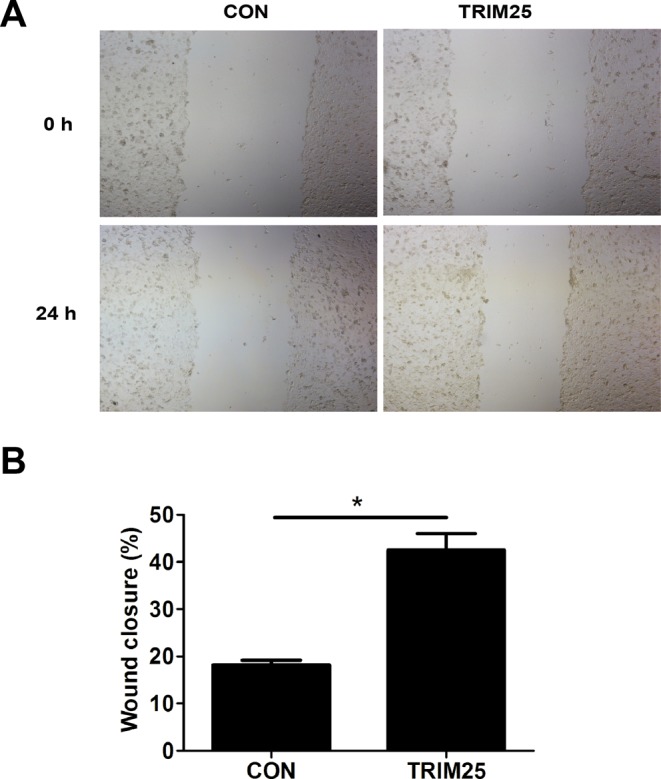
TRIM25 promotes the migration of CRCs. (**A**) Pictures of the scratched area at 0 and 24 h of HCT116 cells stably transfected with either control plasmid (CON) or TRIM25 overexpressing plasmid (TRIM25). (**B**) Migration rate was evaluated by the percentage of wound closure ((initial width − terminal width)/ initial width*100%) according to the images in (A). *P<0.05.

It has been reported that TRIM25 can promote the migration of gastric cancer cells [20]. We therefore tried to figure out whether TRIM25 could promote the migration of CRCs. Wound healing assays were performed to determine the role of TRIM25 in CRCs migration. Scratched cells were photographed under an inverted microscope (Figure 3A). Migration of cells was evaluated by measuring the width of the scratched area at each time point. As shown in Figures 3A,B, overexpression of TRIM25 promotes the migration of CRCs.

### TRIM25 regulates TGF-β signaling pathway in CRCs

**Figure 4:**
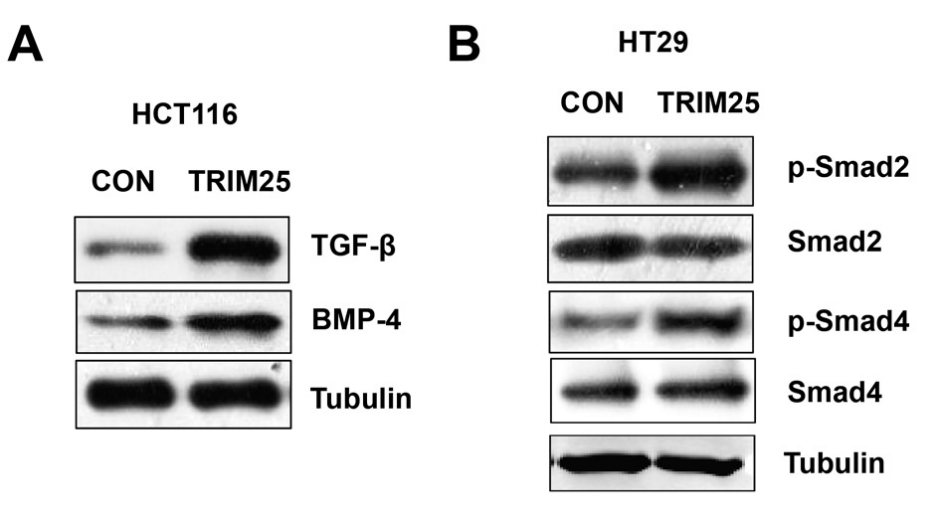
TRIM25 regulates TGF-β signaling pathway. (**A**) Western blot assay of TGF-β, BMP-4, and tubulin of HCT116 cells transfected with either control plasmid (CON) or TRIM25 overexpressing plasmid (TRIM25). (**B**) Western blot assay of p-Smad2, total Smad2, p-Smad4, total Smad4, and tubulin of HT29 cells transfected with either CON or TRIM25.

To identify TRIM25-associated pathways in colorectal cancer, we tested the connection between TRIM25 and TGF-β first, based on a recent report [20]. Western blot results showed overexpressing TRIM25 in HCT116 cells resulted in a significantly increase in TGF-β signaling pathway (TGF-β and BMP-4) (Figure 4A). We also detected overexpressing TRIM25 in HT29 cells increased the phosphorylation of Smad2 and Smad4, which are downstream of TGF-β (Figure 4B). These results indicated that TRIM25 regulates TGF-β signaling pathway in CRCs.

### TRIM25 regulates TGF-β signaling pathway to promote the proliferation and invasion of CRCs

**Figure 5:**
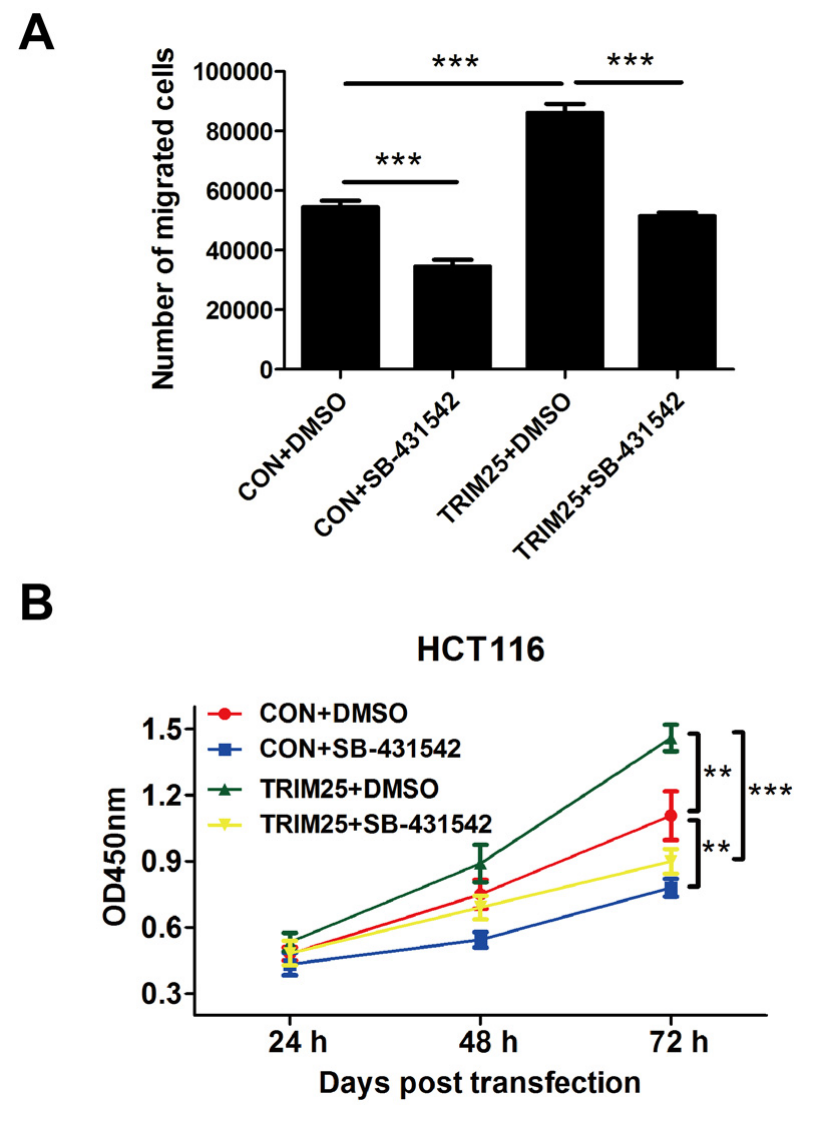
TGF-β inhibitor reverses TRIM25 function on stimulating proliferation and invasion of CRCs. (**A**) The transwell invasion assay was performed on the HCT116 cells transfected with either control plasmid (CON) or TRIM25 overexpressing plasmid (TRIM25). The transwell chamber contained either DMSO or 10 μM TGF-β inhibitor (SB-431542). (**B**) *In vitro* CCK-8 proliferation assay was performed to determine the proliferation rate of HCT116 cells transfected with either CON or TRIM25, and treated with either DMSO or 10 μM TGF-β inhibitor (SB-431542). **P<0.001, ***P<0.0001.

Considering that overexpressing TRIM25 could increase TGF-β level, we supposed that TRIM25 regulates the proliferation and invasion of CRCs through TGF-β signaling pathway. Thus, we performed a transwell invasion assay and a CCK-8 proliferation assay using TGF-β inhibitor SB-431542. Figure 5A showed that cells overexpressing TRIM25 have a significantly higher invasion cell numbers compared with the control cells, but TGF-β inhibitor SB-431542 reversed this stimulation, and the CCK-8 proliferation assay showed the same trend. Cells overexpressing TRIM25 have a significantly higher proliferation rate compared with the control cells; TGF-β inhibitor SB-431542 reversed this stimulation (Figure 5B).

## Discussion

Treatments used for colorectal cancer may include some combination of surgery, radiation therapy, chemotherapy, and targetted therapy. Cancers that are confined within the walls of the colon may be curable with surgery, while cancers that spread widely are usually not curable, with management being directed toward improving quality of life and symptoms. In the United States, the 5-year survival rates are approximately 65%. However, this depends on how advanced the cancer is, whether or not all the cancers can be removed with surgery, and the person’s overall health.

TRIM25 is a member of TRIM proteins and functions as an E3 ubiquitin ligase. TRIM25 has a strong connection with different types of cancers, including breast cancer, ovarian cancer, endometrial cancer, lung cancer, and gastric cancer. It has been reported that TRIM25 has an elevated expression in breast cancer, ovarian cancer, lung cancer, and gastric cancer, but down-regulated in the endometrial carcinoma. The recent studies also revealed that knockdown of TRIM25 suppressed cell growth of lung cancer cells [19] and breast cancer cells [23], but had no effect on the proliferation of gastric cancer cells [20]. All these reports indicate that TRIM25 may have worked as a common oncogene in a broad area of cancers.

However, the knowledge of the expression and possible role of TRIM25 in colorectal cancer is still lacking. In the present study, we found TRIM25 was significantly up-regulated in colorectal cancer tissues and cancer cell lines. When we overexpress TRIM25 in the CRCs, these cells exhibit a significantly higher proliferation and migration rate compared with their parental lines *in vitro*. Moreover, TRIM25 promoted tumor progression *in vivo*. These results showed that TRIM25 also works as an oncogene in the colorectal cancer.

Although the molecular mechanism governing the function of TRIM25 in colorectal cancer was not fully clear, it has been reported previously that TRIM25 positively regulates several cancer-related networks in gastric cancer, including migration, E-cadherin, and TGF-β pathways. It has also been reported that TGF-β is commonly deactivated in colorectal cancers. TGF-β has a deactivating mutation in at least half of colorectal cancers. Sometimes TGF-β is not deactivated, but a downstream protein named SMAD is deactivated [24]. Our study indicated that TRIM25 worked through positively regulating TGF-β signaling pathway to regulate the proliferation and invasion of CRCs.

It has been recognized that cell migration and invasion are important for cancer initiation, progression and metastasis [25]. During tumor progression, epithelial–mesenchymal transition (EMT) is a critical step for the progression of primary tumors toward metastases [26,27]. One of the main factors of EMT is E-cadherin, which contributes to the malignant progression of most carcinomas. Another regulator of EMT is TGF-β signaling pathway. Our study and previous report all showed that TRIM25 regulated both E-cadherin and TGF-β in different types of cancers, suggesting TRIM25 may promote cell migration and invasion through regulating E-cadherin and activating TGF-β signaling pathway, resulting in the regulation of EMT. Further studies will be necessary to explore these possibilities.

As our study showed, TRIM25 activates TGF-β signaling pathway to promote tumor proliferation and metastasis in colorectal cancer. TGF-β signaling pathway controls proliferation, differentiation, and other functions in many cell types. It also plays an important role in controlling the immune system, and shows different activities on different types of cell, or cells at different developmental stages. Based on these functions and the distribution of TGF-β, it is not a good drug target.

TRIM25 only has an elevated expression in either tumor cell lines or tumor tissues, indicating it might be a good drug target candidate. We need to evaluate the TRIM25 expression levels at different stages of colorectal cancers. If TRIM25 expression levels varied at different stages, it might tell us at which stage it is the time to treat patients with target TRIM25 drug, to repress primary tumor growth or inhibit the metastasis. If TRIM25 expression levels keep high in all tumor stages, we may use TRIM25 as a marker to detect patients with colorectal cancers and other TRIM25 associated cancers at a very early stage. If patients with colorectal cancers can be detected at an early stage, it will save most patients’ life through surgery.

Since TRIM25 is not mutated in colorectal cancers, we should do further research to look for the upstream signaling pathway of TRIM25 in colorectal cancers, which stimulates TRIM25 overexpressing and results in the tumor progression. We also need to examine functions of other TRIM family members in colorectal cancer and other types of cancers.

## Conclusion

In conclusion, our results indicate TRIM25 acts as an oncogene in colorectal cancer and it activates TGF-β signaling pathway to promote tumor proliferation and metastasis. Thus, TRIM25 represents potential targets for the treatment of colorectal cancer.

## Abbreviations

BMP: bone morphogenetic protein
CRC: colorectal cancer cell
EMT: epithelial–mesenchymal transition
E3: ubiquitin ligase
RIG-I: retinoic acid inducible gene I
TGF-β: transforming growth factor β
TRIM25: tripartite motif containing 25

## References

[1] Siegel R., Desantis C. and Jemal A. (2014) Colorectal cancer statistics, 2014. CA Cancer J. Clin. 64, 104–117 24639052 10.3322/caac.21220

[2] Miller K.D., Siegel R.L., Lin C.C., Mariotto A.B., Kramer J.L., Rowland J.H. (2016) Cancer treatment and survivorship statistics, 2016. CA Cancer J. Clin. 66, 271–289 27253694 10.3322/caac.21349

[3] Meyerhardt J.A. and Mayer R.J. (2005) Systemic therapy for colorectal cancer. N. Engl. J. Med. 352, 476–487 15689586 10.1056/NEJMra040958

[4] Hayashi M., Inoue Y., Komeda K., Shimizu T., Asakuma M., Hirokawa F. (2010) Clinicopathological analysis of recurrence patterns and prognostic factors for survival after hepatectomy for colorectal liver metastasis. BMC Surg. 10, 10–27 20875094 10.1186/1471-2482-10-27 PMC2949597

[5] Fong Y., Fortner J., Sun R.L., Brennan M.F. and Blumgart L.H. (1999) Clinical score for predicting recurrence after hepatic resection for metastatic colorectal cancer: analysis of 1001 consecutive cases. Ann. Surg. 230, 309–318 10493478 10.1097/00000658-199909000-00004 PMC1420876

[6] Bernassola F., Karin M., Ciechanover A. and Melino G. (2008) The HECT family of E3 ubiquitin ligases: multiple players in cancer development. Cancer Cell 14, 10–21 18598940 10.1016/j.ccr.2008.06.001

[7] Glickman M.H. and Ciechanover A. (2002) The ubiquitin-proteasome proteolytic pathway: destruction for the sake of construction. Physiol. Rev. 82, 373–428 11917093 10.1152/physrev.00027.2001

[8] Mukhopadhyay D. and Riezman H. (2007) Proteasome-independent functions of ubiquitin in endocytosis and signaling. Science 315, 201–205 17218518 10.1126/science.1127085

[9] Schnell J.D. and Hicke L. (2003) Non-traditional functions of ubiquitin and ubiquitin-binding proteins. J. Biol. Chem. 278, 35857–35860 12860974 10.1074/jbc.R300018200

[10] Hatakeyama S. (2011) TRIM proteins and cancer. Nat. Rev. Cancer 11, 792–804 21979307 10.1038/nrc3139

[11] Gack M.U., Shin Y.C., Joo C.H., Urano T., Liang C., Sun L. (2007) TRIM25 RING-finger E3 ubiquitin ligase is essential for RIG-I-mediated antiviral activity. Nature 446, 916–920 17392790 10.1038/nature05732

[12] Gack M.U., Albrecht R.A., Urano T., Inn K.S., Huang I.C., Carnero E. (2009) Influenza A virus NS1 targets the ubiquitin ligase TRIM25 to evade recognition by the host viral RNA sensor RIG-I. Cell Host Microbe 5, 439–449 19454348 10.1016/j.chom.2009.04.006 PMC2737813

[13] Inn K.S., Lee S.H., Rathbun J.Y., Wong L.Y., Toth Z., Machida K. (2011) Inhibition of RIG-I-mediated signaling by Kaposi’s sarcoma-associated herpesvirus-encoded deubiquitinase ORF64. J. Virol. 85, 10899–10904 21835791 10.1128/JVI.00690-11 PMC3187500

[14] Lee N.R., Kim H.I., Choi M.S., Yi C.M. and Inn K.S. (2015) Regulation of MDA5-MAVS antiviral signaling axis by TRIM25 through TRAF6-mediated NF-kappaB activation. Mol. Cells 38, 759–764 26299329 10.14348/molcells.2015.0047 PMC4588718

[15] Urano T., Saito T., Tsukui T., Fujita M., Hosoi T., Muramatsu M. (2002) Efp targets 14-3-3 sigma for proteolysis and promotes breast tumour growth. Nature 417, 871–875 12075357 10.1038/nature00826

[16] Simooka H., Oyama T., Sano T., Horiguchi J. and Nakajima T. (2004) Immunohistochemical analysis of 14-3-3 sigma and related proteins in hyperplastic and neoplastic breast lesions, with particular reference to early carcinogenesis. Pathol. Int. 54, 595–602 15260850 10.1111/j.1440-1827.2004.01668.x

[17] Sakuma M., Akahira J., Suzuki T., Inoue S., Ito K., Moriya T. (2005) Expression of estrogen-responsive finger protein (Efp) is associated with advanced disease in human epithelial ovarian cancer. Gynecol. Oncol. 99, 664–670 16140366 10.1016/j.ygyno.2005.07.103

[18] Nakayama H., Sano T., Motegi A., Oyama T. and Nakajima T. (2005) Increasing 14-3-3 sigma expression with declining estrogen receptor alpha and estrogen-responsive finger protein expression defines malignant progression of endometrial carcinoma. Pathol. Int. 55, 707–715 16271083 10.1111/j.1440-1827.2005.01900.x

[19] Qin Y., Cui H. and Zhang H. (2016) Overexpression of TRIM25 in lung cancer regulates tumor cell progression. Technol. Cancer Res. Treat. 15, 707–715 26113559 10.1177/1533034615595903

[20] Zhu Z., Wang Y., Zhang C., Yu S., Zhu Q., Hou K. (2016) TRIM25 blockade by RNA interference inhibited migration and invasion of gastric cancer cells through TGF-beta signaling. Sci. Rep. 6, 19070 26754079 10.1038/srep19070 PMC4709557

[21] Yang H., Lu X., Liu Z., Chen L., Xu Y., Wang Y. (2015) FBXW7 suppresses epithelial-mesenchymal transition, stemness and metastatic potential of cholangiocarcinoma cells. Oncotarget 6, 6310–6325 25749036 10.18632/oncotarget.3355 PMC4467439

[22] Song L., Xie X., Yu S., Peng F. and Peng L. (2016) MicroRNA-126 inhibits proliferation and metastasis by targeting pik3r2 in prostate cancer. Mol. Med. Rep. 13, 1204–1210 26677064 10.3892/mmr.2015.4661 PMC4732865

[23] Horie K., Urano T., Ikeda K. and Inoue S. (2003) Estrogen-responsive RING finger protein controls breast cancer growth. J. Steroid Biochem. Mol. Biol. 85, 101–104 12943693 10.1016/s0960-0760(03)00209-7

[24] Markowitz S.D. and Bertagnolli M.M. (2009) Molecular origins of cancer: molecular basis of colorectal cancer. N. Engl. J. Med. 361, 2449–2460 20018966 10.1056/NEJMra0804588 PMC2843693

[25] Friedl P. and Wolf K. (2003) Tumour-cell invasion and migration: diversity and escape mechanisms. Nat. Rev. Cancer 3, 362–374 12724734 10.1038/nrc1075

[26] Huber M.A., Kraut N. and Beug H. (2005) Molecular requirements for epithelial-mesenchymal transition during tumor progression. Curr. Opin. Cell Biol. 17, 548–558 16098727 10.1016/j.ceb.2005.08.001

[27] Yang J. and Weinberg R.A. (2008) Epithelial-mesenchymal transition: at the crossroads of development and tumor metastasis. Dev. Cell 14, 818–829 18539112 10.1016/j.devcel.2008.05.009

